# Prevalence of blood stream infections and associated factors among febrile neutropenic cancer patients on chemotherapy at Ocean Road Cancer Institute, Tanzania

**DOI:** 10.1186/s13027-023-00533-8

**Published:** 2023-09-20

**Authors:** Lambert C. Safari, Doreen Mloka, Omary Minzi, Nazima J. Dharsee, Rabson Reuben

**Affiliations:** 1 Department of Clinical Pharmacy and Pharmacy Practice, School of Medicine and Pharmacy, College of Medicine and Health Sciences, University of Rwanda, Huye, Rwanda; 2 University Teaching Hospital of Butare (CHUB), Huye, Rwanda; 3 Department of Clinical Pharmacy and Pharmacology, School of Pharmacy, Muhimbili University of Health and Allied Sciences, Dar es Salaam, Tanzania; 4 Pharmaceutical Microbiology, School of Pharmacy, Muhimbili University of Health and Allied Sciences, Dar es Salaam, Tanzania; 5 Department of Clinical Oncology, School of Medicine, Muhimbili University of Health and Allied Sciences, Dar es Salaam, Tanzania; 6 Ocean Road Cancer Institute, Dar es Salaam, Tanzania

**Keywords:** Bacteremia, BSIs, Susceptibility, Febrile Neutropenia, FN, Cancer, Chemotherapy, Tanzania, ORCI

## Abstract

**Background:**

Febrile Neutropenia (FN) caused by bacteria in cancer patients is associated with poor prognosis. The aim of this study was to determine the prevalence of FN and associated factors among cancer patients on chemotherapy at Ocean Road Cancer Institute (ORCI), Tanzania.

**Methods:**

A cross-sectional study was conducted from June to September 2019. Study participants were conveniently recruited. A desk review of participants medical records was performed. Standard microbiological procedures used to culture and identify the bacterial isolates from the positive blood cultures of participants that presented with FN. Kirby-Bauer disc diffusion was used to perform the antibiotics susceptibility testing. SPSS version 20.0 and MS Excel were used in data entry and analysis. Chi-Square was used as a measure of association between various factors and neutropenia. P-value less than 0.05 was considered statistically significant.

**Results:**

A total 213 participants were enrolled. Of these 76.1% were female. Most of the participants came from the Coast region. Majority of participants presented with breast Cancer (36.2%) and GIT (20.2%). The prevalence of FN and bacteremia was 5.6% and 35.3% respectively. Staphylococcus Aureus (60%) and Coagulase-Negative Staphylococci (40%) were the main isolates. Of the 6 isolates tested most were resistant to Co-Trimoxazole 4/6 (66.7%) and Doxycycline 3/6 (50%). FN was positively associated with chemotherapy regimen (P = 0.0001), platelets count (P = 0.0001) and use of G-CSF (P = 0.0001).

**Conclusion:**

The prevalence of FN among the cancer patients on chemotherapy in Tanzania is low but associated with drug-resistant bacteria.

## Background

Many cancer patients that undergo chemotherapy experience reduced immunity and manifests as low number of neutrophils clinically referred as febrile neutropenia (FN). This is due to the type and intensity of chemotherapy treatment received. Reduced immunity and other risk factors make these patients become more exposed to various infections that may present as chemotherapy-induced febrile neutropenia (CIN) [1].

In cancer patients on chemotherapy, FN is considered as a medical emergency as it is the leading cause of various blood stream infections (BSIs) that require a prompt treatment with the right antibiotics at the right time, which is guided by the current laboratory data. The overall mortality risk rates for cancer patients with FN are 15% times higher compared to cancer patients without FN [2].

In the past (the 1960s and 1970s), gram-negative bacteria were the main etiology of FN. In the early 1980s, gram-positive organisms had become the most commonly isolated pathogens in most cases of FN patients [3]. Nowadays, there seems to be a change in patterns where the most common pathogens being multiple drug resistant (MDR) gram-negative and gram-positive bacteria. Of recent infections with gram-negative MDR bacterial pathogens, including extended spectrum β-lactamase (ESBL)-producing *Enterobacteriaceae,* are particularly prevalent among cancer patients worldwide [4, 5].

In Tanzania, BSIs causative agents in FN cancer patients are unknown and they are often treated empirically without support of current laboratory data with regards to the identity(ies) of the causative agent(s), nor their current antimicrobial susceptibility profiles. This practice puts at risks FN cancer patients to suffer adverse effects of inappropriate antibiotic therapies including death. We therefore embarked on a study to determine the prevalence of bacteremia and antibiotic resistance profile of bacteria in FN cancer patients at Ocean Road Cancer Institute (OCRI).

## Methodology

The aim of this study was to determine the prevalence of FN and associated factors among cancer patients on chemotherapy at Ocean Road Cancer Institute (ORCI), Tanzania.

A cross-sectional hospital and laboratory-based study, was conducted from June to September 2019. All consenting adult cancer patients receiving chemotherapy at Ocean Road Cancer Institute (ORCI) were conveniently recruited. A desk review of the patient’s medical records was conducted to consider patients for inclusion/exclusion into the study and to obtain clinical data including their current clinical data mainly the type of the cancer diagnosed, the stage of the cancer, co-morbidities, type of the chemotherapy, chemotherapy dose being received per cycle, concurrent chemo-radiotherapy, number of the cycles received and the history of antibiotics received. The participants were then interviewed to gain socio-demographic information.

All study participants presenting with FN were drawn two sets of blood samples to detect any blood stream infections (BSI), particularly bacteria.

### Microbiological analysis

A volume of 40 mL was drawn aseptically from participants presenting with FN. The BD BACTEC FX40 automated machine was used to culture blood samples. All positive isolates from Blood cultures were isolated using standard microbiological method and subjected to antibiotic susceptibility testing (AST) using the Kerby-Bauer Disk diffusion as par the CLSI 2018 guidelines criteria.

### Data analysis and quality plan

Data were analyzed using SPSS version 20.0 Descriptive statistics were used present data. Univariate and multivariate regression analysis were used to establish the significance between dependent and independent variables with P-value less than 0.05 which was considered statistically significant.

## Results

### Socio-demographic characteristics

This study recruited 213 participants from different regions of Tanzania. The majority of them were females (76.1%) and 88.7% were married. The majority had a petty business (33.3%). For the performance status, the majority were of ECOG-1 & ECOG-2 (81.9%) (Table 1).

**Table 1:** Study participants’ social and demographic characteristics

Variables	n	%
Gender		
Male	51	23.9
Female	162	76.1
Total	213	100
Age		
< 20	3	1.4
21–30	11	5.2
31–40	33	15.6
41–50	36	17
51–60	55	25.9
61+	74	34.9
Total	212	100
Performance status		
ECOG-0	36	17.1
ECOG-1	94	44.5
ECOG-2	79	37.4
ECOG-3	2	0.9
Total	211	100
Marital status		
Married	189	88.7
Single	16	7.5
Others	8	3.8
Total	213	100
Occupation		
Petty business	65	33.3
Peasants	55	27.4
House wife	40	19.9
Civil servant	23	11.4
Retired servant	12	6
Students	6	3
Total	213	100

ECOG: Eastern Cooperative Oncology Group; Others: Widowed and Divorced

### Clinical characteristics

Among our participants, the mean BP was 125/75 mmHg, mean HR was 85.7 bpm, mean RR was 20.4 rpm, the mean neutrophil count was 45.9% with the mean ANC of 2.4 × 10^3^/µL, and mean Hb of 12.5 g/dL. The mean albumin was 39.8 g/L, mean ALT was 39.8U/L and mean AST was 30.2 U/L. The mean creatinine was 95.2%. 77.8% were over 40 years old (Table 2).

**Table 2:** General clinical parameters for study participants

Units	BP	HR	RR	Age	Neutrophil#	ANC	Platelet	Hb	Albumin	ALT	AST	Creatinine
	mmHg	Bpm	Rpm	Year	%	×10^3^/µL	×10^3^/µL	g/dL	g/L	U/L	U/L	µmol/L
Mean	125/75	85.7	20.4	53	45.9	2.4	284.1	12.5	39.8	24.9	30.2	95.2
Median	122/75	84	20	54	46.8	1.6	272	10.6	39.6	19.3	25	90.9
SD	18.5/10.2	14.2	5.9	14.4	19.4	2.6	190	25.7	10.8	18.72	21.6	32.7
Range	109/58	119	92	75	85.1	20.1	2280.3	365.6	141.5	119.6	144.9	295.5
Min	82/46	15	0	19	2.7	0	0.7	3.4	2.2	1.5	0	1.2
Max	191/104	134	92	94	87.8	20.1	2281	369	143.7	121.1	144.9	296.7
N	213	213	213	213	213	213	213	213	213	213	213	213

BP: Blood Pressure; HR: Heart Rate; RR: Respiratory rate; ANC: Absolute neutrophils count; Hb: Hemoglobin; ALT: Alanine Aminotransferase; AST: Aspartate Aminotransferase, SD: Standard Deviation; Min: Minimum; Max: Maximum

### Prevalence of febrile neutropenia

Out of 213 participants, 14.6% had severe neutropenia, 23.5% had moderate neutropenia, while 9.4% had mild neutropenia. The neutropenia among our study participants was 32.9% with 5.6% presenting with FN (Table 3).

**Table 3:** Prevalence of febrile neutropenia among the study participants

ANC (absolute neutrophils count)	Temperature (°C)				P-value
	≤ 35.0	35.1–37.9	> 38.0	Total	
	n(%)	n(%)	n(%)	n(%)	
Severe neutropenia	0	22(10.3)	9(4.2)	31(14.6)	
Moderate Neutropenia	4(1.9)	43(20.2)	3(1.4)	50(23.5)	
Mild Neutropenia	2(0.9)	18(8.5)	0	20(9.4)	
Normal	1(0.5)	109(51.2)	2(0.9)	112(52.6)	
Total	7(3.3)	192(90.1)	14(6.6)	213(100)	0.0001

### Prevalence of bacteremia

A total of 17 participants presenting with severe neutropenia were each drawn 2 sets of blood samples for culture. The bacteremia rate was 35.3% (6/17) which presented as gas production (5/6) or hemolysis (1/6). Microbiological procedures were performed to identify the isolates from positive culture. All the positive cultures were caused by Gram-Positive bacteria, mainly 66.7% (4/6) were *Staphylococcus Aureus* and 33.7% (2/6) were *Coagulase Negative Staphylococci* (CoNS).

### Bacterial susceptibility testing

We found that 66.7% (4/6) of the isolated pathogens were resistant to SXT 1.25/23.75 µg, 50% (3/6) were resistant to Doxycycline 30 µg, 33.3% (2/6) were resistant to both Imipenem 10 µg and Vancomycin 30 µg and 16.7% (1/6) were resistant to Clindamycin 2 µg. All the isolates were sensitive to cefoxitin 30 µg, gentamycin 10 µg, ciprofloxacin 5 µg, penicillin 10 µg and meropenem 10 µg.

### Association studies

In our study, we have found that various clinical factors were associated with developing neutropenia. By use of univariate and multivariate regression analysis, the following results have been obtained while looking for associations between various factors with neutropenia (Tables 4 and 5). The Performance status, low platelet count, cancer type and the use of G-CSF were found to be statistically significant factors to developing neutropenia.

**Table 4:** Univariate analysis of the factors associated with neutropenia among study participants

		Total	Neutropenic	P-value	95% C.I. for CRUDE OD (lower–upper)
		N(%)	N(%)		
Age	≤ 65.00	172(81.1)	86(86.0)		
	66.00+	40(18.9)	14(14.0)	0.090	0.538 (0.263–1.101)
BMI	< 25	126(60.6)	63(64.9)		
	> 25	82(39.4)	34(35.1)	0.228	0.708 (0.404–1.242)
Performance status (ECOG)	0–1	126(60.9)	72(75.0)	0.000	0.316 (0.174–0.572)
	> 1	81(39.1)	24(25.0)		
Cycle	< 3	115(59.6)	46(53.5)		
	> 3	78(40.4)	40(46.5)	0.123	1.579 (0.884–2.820)
Hemoglobin	< 10	81(38.2)	42(42.0)		
	> 10	131(61.8)	58(58.0)		0.738 (0.423–1.286)
Platelet counts	< 150	43(20.2)	34(33.7)	0.000	
	151–449	148(69.5)	59(58.4)	0.001	0.151 (0.048–0.472)
	> 450	22(10.3)	8(7.9)	0.754	0.862 (0.340–2.182)
Cancer type	Hematological	31(14.6)	25(24.8)	0.000	0.172 (0.067–0.440)
	Non-hematological	182(85.4)	76(75.2)		
Heart Rate	< 60	5(2.4)	4(4.0)	0.132	
	60–100	186(87.7)	83(83.0)	0.455	0.406 (0.038–4.310)
	> 100	21(9.9)	13(13.0)	0.138	2.017 (0.798–5.095)
Gender	Male	51(23.9)	24(23.8)		
	Female	162(76.1)	77(76.2)	0.953	1.019 (0.543–1.914)
Albumin	< 35	44(21.2)	23(23.2)	0.779	
	35–55	162(77.9)	75(75.8)	0.95	0.913 (0.054–15.538)
G_CSF	Yes	84(40.0)	62(61.4)		
	No	126(60.0)	39(38.6)	0.000	0.159 (0.086–0.294)

**Table 5:** Multivariate analysis of various factors

Variables		P-value	OR	95% C.I. for EXP (B)	
				Lower	Upper
Performance Status (ECOG)	0–1				
	> 1	0.017	0.425	0.210	0.859
Platelets counts	< 150	0.006			
	151–449	0.013	0.185	0.048	0.704
	> 450	0.608	0.747	0.245	2.276
Cancer type	Hematological				
	Non hematological	0.139	0.447	0.154	1.297
G_CSF	Yes				
	No	0.000	0.216	0.110	0.425

## Discussion

**Fig. 1:**
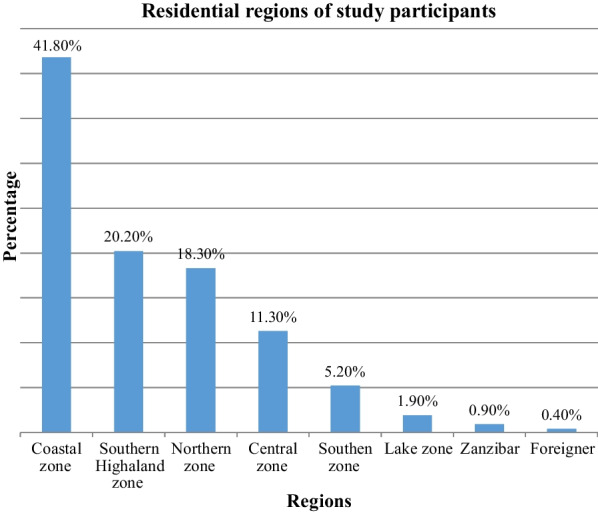
Regional distribution of study participants

Bacteremia is a common blood stream infection among febrile neutropenic cancer patients on chemotherapy. If not treated urgently and adequately with an appropriate antibiotic, the worst adverse complication happens to the patient: death. In this study, we found that that females (76.1%), as shown in previous studies in Tanzania, have a higher cancer health seeking behavior than males [6]. The finding that Lake Zone have the least number of participants as compared to Dar es Salaam and coastal regions, could be attributed to that most patients from lake zone currently consult at their nearest hospital, Bugando Medical Center (BMC), which has recently started offering cancer services while others may travel miles from their homes to the Coast Region to seek shelters where they can stay while seeking the health care services at ORCI [7] (Fig. 1).

**Fig. 2:**
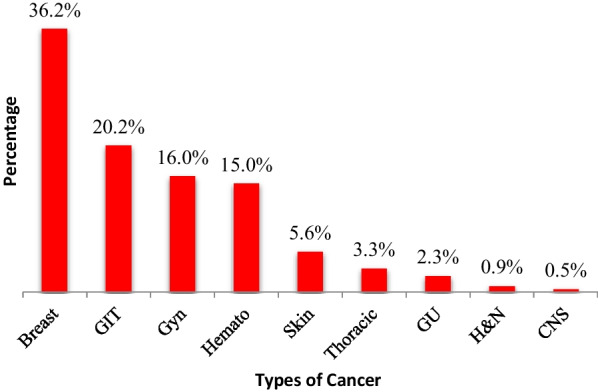
Types of cancer among study participants. GIT: Gastro-Intestinal Tract; Gyn: Gynecological; Hemato: Hematological; GU: Genito-Urinary; H&N: Head &Neck; CNS: Central Nerve System

The breast cancer (36.2%) and Gastro-Intestinal tract (GIT) cancers (20.2%) were the most prevalent in our study. This may be due to that breast cancers and gynecological cancers are the most prevalent in Tanzania. Moreover, it could be from the fact that they are more readily detected as the Government has provided facilities and supports regular screening for these type of cancers, adding that women have more seeking behaviors for health care services than men [7] (Fig. 2).

The majority of participants had the performance status of ECOG-2. The main reasons for this could be that most of the patients consult health care services late while some of them seek first the traditional healer or stay home and come for consultation when the disease has already progressed [6]. The practice of delayed health seeking behavior of healthcare services could be a result of low awareness of cancer especially among women from rural areas of Tanzania [8, 9]. The other reasons could be that patients who come from faraway are afraid of the financial burden to the patients and their families as well as the stigma associated with cancer [10, 11].

**Fig. 3:**
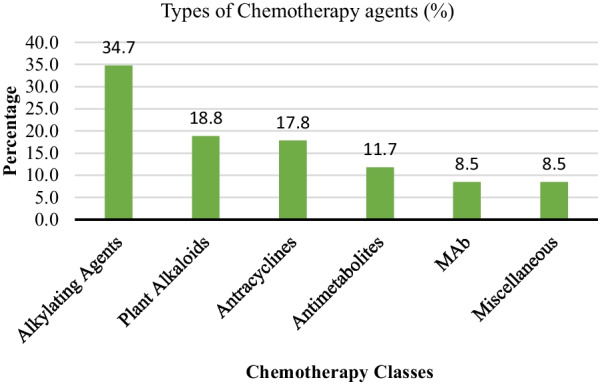
Types of Chemotherapy agents used by study participants

The overall neutropenia rate was 38.1%, with 23.5% presenting as moderate neutropenia and 14.6% as severe neutropenia. This neutropenia rate is high, in our settings and factors like age, performance status, G-CSF as part of their treatment regimen, low platelet count and the type of cancer, mainly the hematological cancer are likely to induce neutropenia. The severe neutropenia rate was high in study participants below 20 years of age is in contrast to findings from other studies, which have linked severe neutropenia with older age [3, 4], but this could be linked with the hematological cancers that were the most prevalent in this young age group. These findings need to be further investigated, particularly if the neutropenia is associated with viral and parasitic pathogens for which we were not able to identify. However, our findings are similar to other studies in others countries that have linked development of severe neutropenia with hematological cancer, and older age [3, 4]. The rate of severe neutropenia was also high in people using alkylating agents, anthracyclines, imatinib and antimetabolites as part of their chemotherapy regimen (Fig. 3). These chemotherapeutic classes are known commonly to cause neutropenia [12]. However, participants using plant alkaloids had a low severe neutropenia rate in comparison to other chemotherapy agents. In 2009, Pettengell et al. [13] have found neutropenia rate in cancer patients to be 35% in a study done in Europe and this was similar to what we found in our study despite the difference in settings and facilities. Nonetheless, in the present study, neutropenia rate is much lower than those of studies conducted in Japan where the Chemotherapy-induced neutropenia was found to be 50.5% [14]. The practices, environmental factors and local settings maybe the main key contributors to findings despite the difference in settings. In Tanzania, there a common practice of administering the G-CSF to patients who present with moderate and severe neutropenia to boost their immunity and to keep the track in their treatment courses.

The prevalence of FN was 5.6% in the general population. It was low because many patients getting neutropenia are administered the G-CSF and this may delay the anticipated FN, which is beneficial to the patients attending chemotherapy ward at ORCI. The use of G-CSF may contribute to the prevention of FN among the cancer patients receiving chemotherapy and also enhancing to boost their immunity while achieving the desired therapeutic outcomes. However, our findings have found a likelihood contribution of the use of these G-CSF in developing neutropenia. They contribute to the accelerated myelosuppression which later leads to the lack of immunity to the patients, hence the increased susceptibility to blood stream infections. Comparing to other studies in other settings, there is a close similarity of results with those of Japan and USA [15]. Contrary, the FN rates were low in our settings compared to those in other countries around the world [16–21]. This may show the improved practices in Tanzania while managing the FN among this group of patients.

The prevalence of bacteremia was very high and mainly caused by gram-positive bacteria. The most common isolates were *S. Aureus* and were associated with resistance to commonly prescribed antibiotics. This is something alarming in Tanzania among the cancer patients on chemotherapy, as they may be prone to non-curable infections or expensive to treat as well. The isolates found in our results were similar to what found in other studies conducted around the globe which presented with alarming findings [4, 22, 23]. However, the bacteremia rates are higher than those of other studies conducted in other countries around the world where the prevalence of bacteremia reported in those countries was lower than that we found in our settings [23–29]. This variability in terms of prevalence may have been a result of our limited sample size for this study, and the study design but also the study settings available.

The bacterial isolates were found to be resistant to some strong antibiotics like vancomycin and meropenem, but also sensitive to Oxacillin (Cefoxitin). The staphylococci, mainly *S. Aureus* were the most prevalent among the participants and majority of them were MDR. In our study settings, attention is needed as the infections are caused by Gram-Positive bacteria that drug-resistant; and the empirical choice of the antibiotics should be based on the individual patient blood culture and AST data. Otherwise, the AST for any admitted FN is needed to accurately provide the right antibiotics. Studies conducted in Egypt and Iran had similar results to ours in that the majority of infections among the FN cancer patients on chemotherapy were caused by Gram-Positive Bacteria [28]. *Staphylococcus Aureus* (S.A) = 67% and *Coagulase-Negative Staphylococci* (CoNS) = 33% members of the ESKAPE group were the main isolates of bacteria of the study. Moghnieh et al. 2015 reported similar isolates in Lebanon and South Africa where studies by Louw et al. 2010 found that 42.7% and 49% of bacteremia in FN cancer patients were caused by gram-positive bacteria (GP), specifically methicillin-resistant CoNS [12, 16]. The finding that only gram-positive bacteria were associated with bacteremia in Tanzanian FN cancer patients is in contrast to findings from other studies that associated bacteremia in FN cancer patients with both gram-positive and gram negative bacteria [4, 30–32]. This variability may be due to limited number of repeated samples taken to perform blood cultures in this study. As studies have shown that repeating blood cultures may increase the success rate of detecting positive blood cultures [33–36].

In terms of antibiotics susceptibility patterns all isolates were 100% sensitive to cefoxitin, gentamycin, ciprofloxacin, penicillin and meropenem, and which is similar to what was found in studies done in other countries like USA, India, Iran, Ghana and Uganda [23, 37–42].

Nevertheless, our study found that the majority of isolates to have a high resistance rate to SXT and Doxycycline, with a moderate resistance to Imipenem, Vancomycin and Clindamycin. And similar findings have reported in Uganda which is a neighbor Country for Tanzania [37]; proving that the AMR problem is real in the Sub-Saharan Africa. The isolation of multi-drug resistance (MDR) *Staphylococcus aureus* is a warning signal to our clinical settings, as this finding suggests that the antimicrobial resistance (AMR) may be increasingly being evidenced among cancer patients. The emergence of MDR pathogens causing FN in cancer patients in Tanzania indicates treating FN cancer may be a challenge in future due to wide spread AMR as it has been shown in other countries [22, 43, 44]. Moreover, the finding that resistance to Vancomycin was 50% of isolated CoNS and in 25% of isolates *S. Aureus* may be an indicator that antimicrobial stewardship and rational use of antimicrobials needs to be strengthened in Tanzania [14, 17–20, 45]. The current practice of clinicians prescribing without support of microbiology laboratory AST data needs to be curbed through continuing professional development and advocating for antimicrobial stewardship in clinical settings. However, enforcing antimicrobial stewardship in LMICs like Tanzania, maybe a challenge due to limited resources for health. To effectively use the available health care resources in Tanzania, Policy-makers have to improve the existing policies, guidelines and practices for antimicrobial stewardship. This will ensure reduced mortality, morbidity and cost associated with treating FN in cancer patients infected with MDR pathogens and continued to use Ciprofloxacin for prophylaxis [21, 46, 47].

Several factors were associated with developing neutropenia. With the univariate and multivariate regression analysis, we found that the type of cancer (Hematological Vs Non-hematological), low platelet count, use of G-CSF and poor performance status of the patient can be the predictive factors for likelihood of developing neutropenia and eventually FN. There was poor performance status [15] and the same finding have been reported in Canada by Younus [48]; however, there was no association of neutropenia with combined therapy in our study as it has been reported in Japan and United Kingdom (UK) [15, 48] as was reported in the UK [49]. Differences in patient populations may have contributed to these differences. However, we were unable to do the correlation analysis between neutropenia and bacteremia as well as between bacteremia and type of tumor because bacteremia status was found in a few patients (6/17). We hope that other studies will address this correlation in the future.

### Study limitations

There was a risk of missing the real candidates who presented with FN because some of them were quickly admitted and administered antibiotics as well as the G-CSF. Those candidates would be found at the time when it is not possible to draw the blood for culture because it could lead to false results. We were unable to identify the fungal infections as well.

## Conclusion

The prevalence of FN among the cancer patients on chemotherapy is relatively low at ORCI compared to other countries. The use of G-CSF, low platelet count and poor performance status have been found to contribute to developing neutropenia among this group of patients. However, bacteremia rates are relatively higher and associated with drug resistant pathogens. Clinicians at OCRI should consider the previous regimen of the patient as well as the performance status to predict the likelihood of cancer patients developing neutropenia, that may eventually lead to developing FN. There should be a regular test for blood culture and AST for effective patient management.

## Data Availability

The datasets used and/or analyzed during the current study are available from the corresponding author on reasonable request.

## Abbreviations

AIDS: Acquired Immuno-Deficiency Syndrome
ALT: Alanine Aminotransferase
AMR: Antimicrobial resistance
ANC: Absolute Neutrophil Count
ASP: Antimicrobial Stewardship Program
AST: Antibiotics susceptibility testing
BMC: Bugando Medical Center
BMI: Body Mass Index
BSIs: Blood Stream Infections
CNS: Central Nervous System
CoNS: Coagulase-negative Staphylococci
COPD: Chronic Obstructive Pulmonary Disease
CPL: Central Pathology Laboratory
CSF: Colony stimulating factor
DBP: Diastolic Blood Pressure
ECOG: Eastern Cooperative Oncology Group
ESKAPE: Enterococcus faecium, Staphylococcus aureus, Klebsiella pneumoniae, Acinetobacter baumannii, Pseudomonas aeruginosa and *Enterobacter* spp.
ESBL: Extended Spectrum β-lactamase producing Enterobacteriaceae
FAO: Food and Agriculture Organization
FBP: Full blood picture
FN: Febrile Neutropenia
GARP: Global Antimicrobial resistance partnership
G-CSF: Granulocyte-colony stimulating factor
GIT: Gastro-Intestinal Tact
GN: Gram Negative
GP: Gram Positive
GU: Genito-Urinary
HIV: Human Immune Virus
H&N: Head and Neck
HR: Heart rate
HTN: Hypertension
IDSA: Infectious disease society of America
IV: Intra-Venous
LMICs: Low- and Middle-Income Countries
MAb: Monoclonal antibody
MBC: Metastatic Breast Cancer
MCRC: Metastatic Colon/Rectal Cancer
MLC: Metastatic Lung Cancer
MNH: Muhimbili National Hospital
MOC: Metastatic Ovarian Cancer
MPC: Metastatic Prostate Cancer
MRSA: Methicillin Resistant Staphylococcal Aureus
MUHAS: Muhimbili University of Health and Allied Sciences
ORCI: Ocean Road Cancer Institute
PMN: Poly-Morph Nuclear cells
RR: Respiratory rate
SBP: Systolic Blood Pressure
SD: Standard Deviation
ST: Soft Tissue
SXT: Sulfamethoxazole/Trimethoprim
UICC: Union for International Cancer Control
WHO: World Health Organization
